# Research on lung nodule recognition algorithm based on deep feature fusion and MKL-SVM-IPSO

**DOI:** 10.1038/s41598-022-22442-3

**Published:** 2022-10-18

**Authors:** Yang Li, Hewei Zheng, Xiaoyu Huang, Jiayue Chang, Debiao Hou, Huimin Lu

**Affiliations:** 1 School of Computer Science and Engineering, Changchun University of Technology, Changchun, 130012 Jilin China; 2 School of Computer Science and Engineering, Jilin University of Architecture and Technology, Changchun, 130114, Jilin China

**Keywords:** Biomedical engineering, Classification and taxonomy, Image processing, Learning algorithms, Network models

## Abstract

Lung CAD system can provide auxiliary third-party opinions for doctors, improve the accuracy of lung nodule recognition. The selection and fusion of nodule features and the advancement of recognition algorithms are crucial improving lung CAD systems. Based on the HDL model, this paper mainly focuses on the three key algorithms of feature extraction, feature fusion and nodule recognition of lung CAD system. First, CBAM is embedded into VGG16 and VGG19, and feature extraction models AE-VGG16 and AE-VGG19 are constructed, so that the network can pay more attention to the key feature information in nodule description. Then, feature dimensionality reduction based on PCA and feature fusion based on CCA are sequentially performed on the extracted depth features to obtain low-dimensional fusion features. Finally, the fusion features are input into the proposed MKL-SVM-IPSO model based on the improved Particle Swarm Optimization algorithm to speed up the training speed, get the global optimal parameter group. The public dataset LUNA16 was selected for the experiment. The results show that the accuracy of lung nodule recognition of the proposed lung CAD system can reach 99.56%, and the sensitivity and F1-score can reach 99.3% and 0.9965, respectively, which can reduce the possibility of false detection and missed detection of nodules.

## Introduction

According to the GLOBOCAN2020 data released by International Agency for Research on Cancer (IARC), lung cancer remained the leading cause of cancer death. In 2020, about 1,796,144 people died of lung cancer, accounting for 18% of all cancer deaths^1^. Early screening of lung cancer is an effective method to reduce mortality, which can improve the 5-year survival rate of patients from 18.6% to 56%^2^. Lung nodules are an early manifestation of lung cancer. Due to the high resolution of high-density tissue, computed tomography (CT) imaging has become an essential means of detecting and identifying lung nodules^3–5^.

However, with the increase in the number of lung cancer patients and people’s emphasis on health in recent years, and a large number of CT images have been produced clinically, which has brought enormous pressure to doctors. In addition, there are differences in the diagnosis and treatment level of doctors with different seniority, so for the same CT image, different doctors are likely to give different diagnostic results. Lung computer aided diagnosis (CAD) system can assist doctors in obtaining objective diagnostic results and effectively reduce missed detection and false detection of nodules^6^. The classic lung CAD system usually includes the following steps: image preprocessing, lung parenchyma segmentation, lung nodule candidate region of interest (ROI) extraction, ROI feature extraction and lung nodule recognition. Among them, feature extraction and lung nodule recognition are the core modules of lung CAD system, which will directly affect the performance of the system^7^.

## Related work

Feature extraction is an essential link in the lung CAD system. Traditional lung CAD systems mainly rely on the experience of doctors, extract handcrafted features of low-level vision, such as texture information and morphological brightness of lung nodule images, and then input a machine learning-based classifier for recognition^8,9^. Traditional lung CAD systems need to design handcrafted features in advance, and the extracted handcrafted features cannot express the high-level semantic information of nodules, resulting in poor generalization ability of the model.

In recent years, deep learning has become the mainstream method for medical image feature extraction^10^. Deep learning can capture every detail in the image. It can extract different levels of features from different depth layers, which is more suitable for the analysis and processing of medical images. Among them, the Convolutional Neural Network(CNN) has the widest application range due to its excellent performance^11–13^. Deep learning is a representation learning algorithm based on large dataset, but labelled medical imaging datasets are often scarce^14^.

Transfer learning is a method of transferring knowledge from the source domain to the target domain. It does not require that the training data be independent and identically distributed with the test data, which provides a possibility to solve the problem mentioned above of insufficient labelled training data in medical image processing^15^. Currently, fine-tuning and feature extraction is the most commonly used transfer learning strategies in medical image analysis^16^. The fine-tuning uses the parameters of the source domain model as the initialization parameters, migrates to the target domain model, and then uses the medical imaging dataset to fine-tune the initialization parameters^17,18^. The other is to remove some layers from the model pre-trained in the source domain as the feature extractor of the target domain. Then add another classifier to rebuild a new network model^19,20^.

Using deep learning methods to extract features usually takes the entire image as input. However, medical image analysis pays more attention to the focus area information, and the global processing of the image will lead to information redundancy. As an efficient resource allocation scheme, attention mechanism has been applied to the feature extraction model^21^. Embedding the attention mechanism into the neural network can guide the network to focus on the important information of the lesion area in a high-weight manner, and ignore irrelevant information in a low-weight manner, thereby improving the feature extraction ability of the network^22–24^.Sun et al.^22^ proposed the attention-embedded complementary-stream convolutional neural network (AECS-CNN) to reduce false positives of lung nodules. AECS-CNN employed two convolutional block attention module (CBAM) to weight multi-scale features, and then assign higher weights to key features to improve the recognition sensitivity of lung nodules to 92%. Wang et al.^24^ proposed a novel network for Alzheimer’s disease recognition. The network was modified on the basic framework of VGG16, embedded CBAM after each convolution block, and the final accuracy reached 97.76±1.13%.

The features extracted by a single model can reflect image information to a certain extent, but some information is missing, which has limitations^25^. Feature fusion can derive a more low-dimensional and effective feature vector set in multiple feature sets and benefit to the final decision^26^.The classic feature fusion strategies are serial fusion and parallel fusion^27–29^.However, these two fusion strategies simply splicing feature vectors, and the dimension of the fused feature set is high, which is prone to problems such as dimensional disaster. Another feature fusion strategy is to first map different types of feature vectors to a new dimensional space, and then fuse them into new features. This fusion method fully exploits the relationship between features and combines them in a new projection space, which not only reduces the feature dimension, but also removes redundant features. The representative algorithms are canonical correlation analysis (CCA) based on projective transformation, feature fusion based on sparse representation^30,31^. Among them, CCA can generally grasp the correlation between the two sets of feature sets, and is often used for feature fusion between the two sets of feature sets^32,33^.Kiran et al.^34^ used CCA to fuse the global average pooling (GAP) layer of Resnet-50 and the deep features of fully connected layer(FCL) to achieve Human Action Recognition (HAR). Peng et al.^35^ used CCA to fuse the deep features of different networks to obtain more discriminative fusion features, thereby improving the recognition accuracy of grape species.

Nowadays, there are mainly three models in image recognition tasks. The first is the classic lung CAD system, which feeds handcrafted features into traditional machine-based classifiers; the second is called solo deep learning (SDL) model, which runs through the entire process in an “end-to-end” manner. The third is the hybrid deep learning (HDL) model, which integrates various traditional machine-based deep learning-based feature extraction learning classifiers for cascading presentation, thereby flexibly improving the model’s classification performance^36^.

In image recognition or classification, the CAD system based on HDL is mainly divided into two stages: feature extraction and image recognition or classification.

In the first stage of HDL, deep features are usually extracted using transfer learning techniques combined with classic CNN models. In order to reduce the false positive rate of lung nodules, Shi et al.^37^ used the fine-tuned VGG16 model to extract deep features, and input the support vector machine (SVM) to identify whether the candidate ROI was a nodule. Finally, 87.8% accuracy and 87.2% sensitivity were obtained on LIDC-IDRI. Mastouri et al.^38^ used the pre-trained bilinear CNN model as the feature extractor, and combined with SVM, AdaBoost, k-Nearest Neighbor (KNN), random forest and FCL to distinguish nodules or non-nodules. The results on LUNA16 showed that the bilinear CNN model composed of VGG16 and VGG19 as a feature extractor combined with SVM has the best effect on lung nodule recognition, and the accuracy can reach 91.99%. Khan et al.^39^ used the pre-trained VGG-19 supported segmentation (VGG-SegNet) extract the lung nodule section, concatenated deep and handcrafted features and fed into a classifer to classify lung nodules. Compared with softmax, Decision Tree RF and KNN, SVM-RBF achieved the highest accuracy up to 97.83%.

In the second stage of the HDL model, the classifier based on traditional machine learning can obtain better recognition or classification performance than the FCL^40^. However, the conventional machine learning algorithm has disadvantages such as a large amount of calculation and a long time to find the optimal parameter group in the training process. Swarm intelligence optimization algorithm provides convenience for fast optimization of parameters. The classic swarm intelligence optimization algorithms mainly include ant lion algorithm^41^, genetic algorithm (GA)^42^, particle swarm algorithm (PSO)^43^and so on. Poap et al.^44^used neural networks combined with ant lion algorithm for lung disease detection. Although the ant-lion algorithm has the advantages of simplicity and high convergence accuracy, it also has a serious problem of relying on elite ant-lions, which increases the possibility of falling into a local optimum, which makes the algorithm prone to premature convergence^45^. As a typical representative of swarm intelligence optimization algorithm, PSO is widely used in parameter optimization due to its simple concept and easy implementation with few parameter settings^46^. Balaha et al.^47^ proposed a hybrid deep learning and genetic algorithm approach for early ultrasound diagnosis of breast cancer. Different from the GA, PSO does not need to go through steps such as crossover, mutation, evolution, etc., avoiding complex evolutionary operations. It adopts mobile search in the entire global environment, and can adjust its search strategy at any time according to the current situation. Because all particles are constantly moving and changing during the search, it is an efficient parallel search strategy^48^.However, the standard PSO algorithm has the problems of being sensitive to parameters, prone to fall into local optimal solutions due to premature particles, and slow convergence^49^.Wang et al.^50^ proposed a support Vector machine algorithm of particle swarm Optimization based on an adaptive mutation to realize cell swarm identification. The correctness can reach 99.79%. Li et al.^51^ combined the Radial Basis Function (RBF) and the polynomial kernel function into a multi-kernel function in the form of linear convex combination to form MKL-SVM to realize lung nodule recognition. To overcome the problem of too long parameters optimization time, the PSO was combined with the Multi Kernel Learning Support Vector Machine (MKL-SVM) algorithm. Furthermore, to obtain the global optimal solution, constant inertia weight, linear inertia weight and nonlinear inertia weight were respectively used to improve PSO. In the end, found a more effective nonlinear inertia weight, and the recognition accuracy on the test set can reach 91%. The difference between this paper and the literature^51^ is that the proposed improved Particle Swarm Optimization(IPSO) algorithm uses different inertia weights, which are adaptive inertia weights. By adopting corresponding weight adjustment strategies for particles in different subgroups, the particles can be optimized adaptively. , and the dynamic learning factor is further introduced to adjust the self-learning ability and collective learning ability of particles.

Based on the HDL model, this paper focuses on three critical algorithms of the lung CAD system: feature extraction, feature fusion and nodule recognition. Aiming to improve the accuracy and sensitivity of lung nodule recognition. The main contributions are as follows:

1. Propose a deep feature extraction model with embedded attention mechanism Attention-embedded VGG16 (AE-VGG16) and Attention-embedded VGG19 (AE-VGG19). The model first embeds the CBAM into the classic CNN models VGG16 and VGG19, respectively. This way the network can pay more attention to the key points in the nodule description. Then uses the parameters of the pre-trained model on ImageNet as initialization parameters, and retrain the weights with the preprocessed candidate nodule images to reduce the training cost and supplement the information loss in the target domain.
2. Feature fusion using CCA. The feature vector set with solid correlation between feature groups is selected as the fused feature. The fusion feature has powerful feature expression ability and low-dimensional characteristics, which not only reduces the amount of calculation, but also helps to improve the subsequent recognition effect.
3. A Multiple Kernel Learning Support Vector Machine based on the improved Particle Swarm Optimization algorithm (MKL-SVM-IPSO) was proposed for lung nodule recognition. Using the same MKL-SVM as^51^, and introducing the adaptive inertia weight particle swarm optimization algorithm PSO, according to the fitness value adaptive fast parameters Optimization to speed up the training of the model. The dynamic learning factor is further adapted to adjust the self-learning ability and collective learning ability of particles. It solves the problem that the model training speed is slow, and easy to fall into the local optimum.

The novelty of our method is that this method improves the feature extraction ability of the network by combining the attention mechanism with CNN, and then uses the feature dimension reduction technology to reduce the deep features of tens of thousands of dimensions to less than 100 dimensions. Under the premise of the amount of information, the calculation amount of the model is reduced. Feature fusion through CCA can mine the correlation between features, and obtain fusion features with strong correlation and more conducive to nodule identification. Finally, the intelligent optimization algorithm is combined with MKL-SVM, which solves the problem of slow model training and easy to fall into local optimum, and further improves the performance of the system.

## Materials and methods

### Experimental dataset

All experiments were performed in accordance with relevant named guidelines and regulations, with informed consents obtained from all subjects. The LUNA16 dataset (https://luna16.grand-challenge.org/Data/ established by the NIH and NCI of the United States) is used to train and test the proposed model^52^. This dataset is freely available to browse, download, and use for commercial, scientific and educational purposes as outlined in the Creative Commons Attribution 4.0 International License. The DeepLesion dataset that support the findings of this study are openly available at (https://nihcc.app.box.com/v/DeepLesion published by the NIH Clinical Center)^53^.

The experiments are performed on the public dataset LUNA16^52^. LUNA16 collected 888 low-dose lung CT images from the LIDC-IDRI, filtered out scans with slice thicknesses greater than 2.5 mm, including 1186 nodules marked by radiologists.

**Figure 1:**
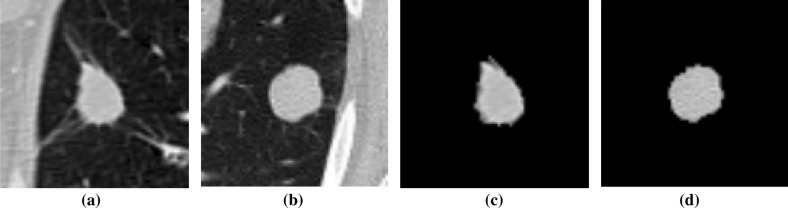
Preprocessing process of positive samples.

Before feature extraction, a image preprocessing operation is required. In the existing research of image enhancement algorithms, the histogram equalization algorithm is widely used because of its advantages of simple and fast calculation^54^. The basic principle is to extend the dynamic range of an unevenly distributed image histogram to both sides, making it even, thereby improving the overall contrast of the image. However, in order to enhance the entire image, if there is a large area of low gray level in the image, the result of the enhancement will be too bright and the contrast is not obvious enough. The grayscale distribution in CT images of the lungs is usually relatively concentrated, making the lung nodule site look unclear and increasing the difficulty of extracting ROI. So our image preprocessing steps are as follows. The preprocessing process for positive sample images is as follows: First, frame the lesion area containing lung nodules according to the annotation information given by the doctor, place the nodule part in the center of the image, and crop out an image with a size of 64*64. Then 650 images of solitary nodules were screened, the original image of the lesion area is shown in Fig. 1a, b. Finally, the cropped image is binarized. In order to eliminate the background interference, the largest 8 connected regions are reconstructed. The maximum entropy method is used to select the threshold, and finally the ROI image of the lung nodule is obtained, the corresponding preprocessed positive sample images are shown in Fig. 1c,d.

**Figure 2:**
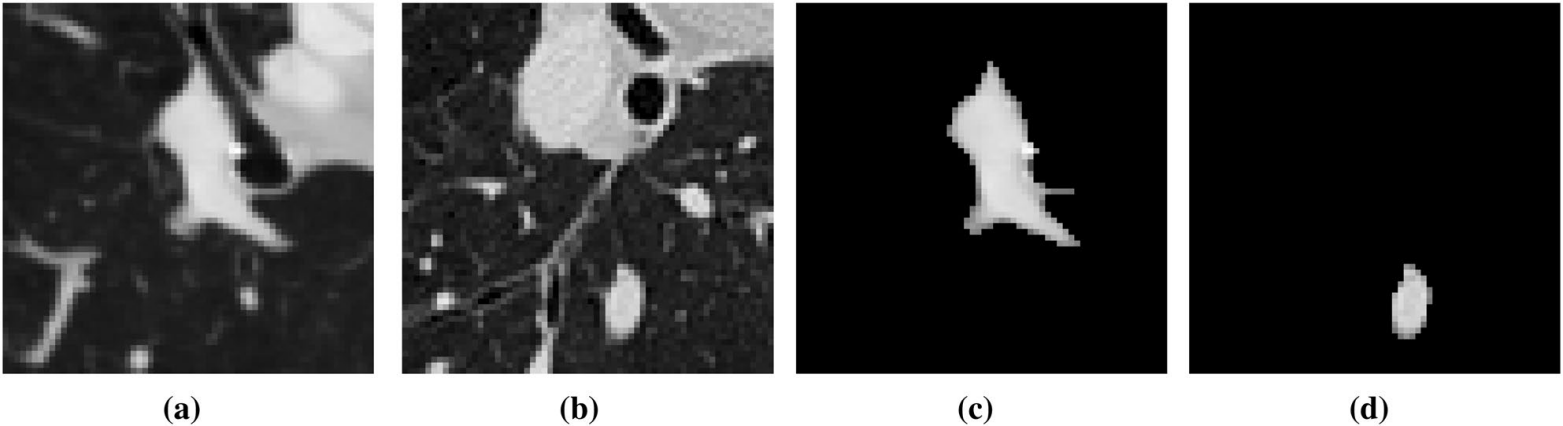
Preprocessing process of negative samples.

The preprocessing process for negative sample images is as follows: first, the lesion area is filtered out, and in the area without any lesions, the suspected lesion area containing tissue or blood vessels similar in shape to nodules is selected, and an image with a size of 64*64 is randomly intercepted. After the same process as making positive samples, 490 non-nodule images were finally selected as negative samples .The original image of the lesion area is shown in Fig. 2a,b. The corresponding preprocessed negative sample images are shown in Fig. 2c, d.

After image preprocessing consistent with the literature^55^, 1140 ROI images of candidate nodules with a size of 64*64 were selected as the experimental dataset. Specifically, 650 images of solitary lung nodules and 490 non-nodule images were included. After randomly shuffling the dataset, 912 and 228 images were selected as training sets and test sest according to the ratio of 8:2.

### Proposed lung CAD system

**Figure 3:**
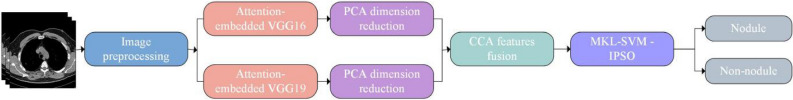
The block diagram of the proposed lung CAD system.

The proposed lung CAD system specifically includes the following five main steps: preprocessing of lung CT images, feature extraction based on attention mechanism and transfer learning technology, feature dimensionality reduction based on Principal component analysis (PCA), feature fusion based on CCA and lung nodule identification based on MKL-SVM-IPSO algorithm. A detailed description of the key algorithms of the improved lung CAD system is provided below. The block diagram of the proposed lung CAD system is shown in Fig. 3.

### Feature extraction based on AE-VGGNet

VGGNet is selected as the basic model of the feature extraction model, the motivation is that this model is particularly suitable for transfer learning and has good feature extraction ability^56^. In addition, VGGNet has also been shown to have excellent generalization ability and can adapt to other domain images except ImageNet dataset with good performance^57^. In recent years, many scholars have fused the features of VGG16 and VGG19 to improve the performance of the model^58–60^. In terms of network architecture, VGG19 has three more convolutional layers than VGG16, so the feature semantic information extracted by VGG19 is richer, and the detailed information contained in VGG16 is more comprehensive. In order to combine the advantages of the two, CCA is used for feature fusion, and only the feature vectors with strong correlation between the two sets of feature vectors are selected to obtain more low-dimensional and effective fusion features that are beneficial to the final decision.

**Figure 4:**
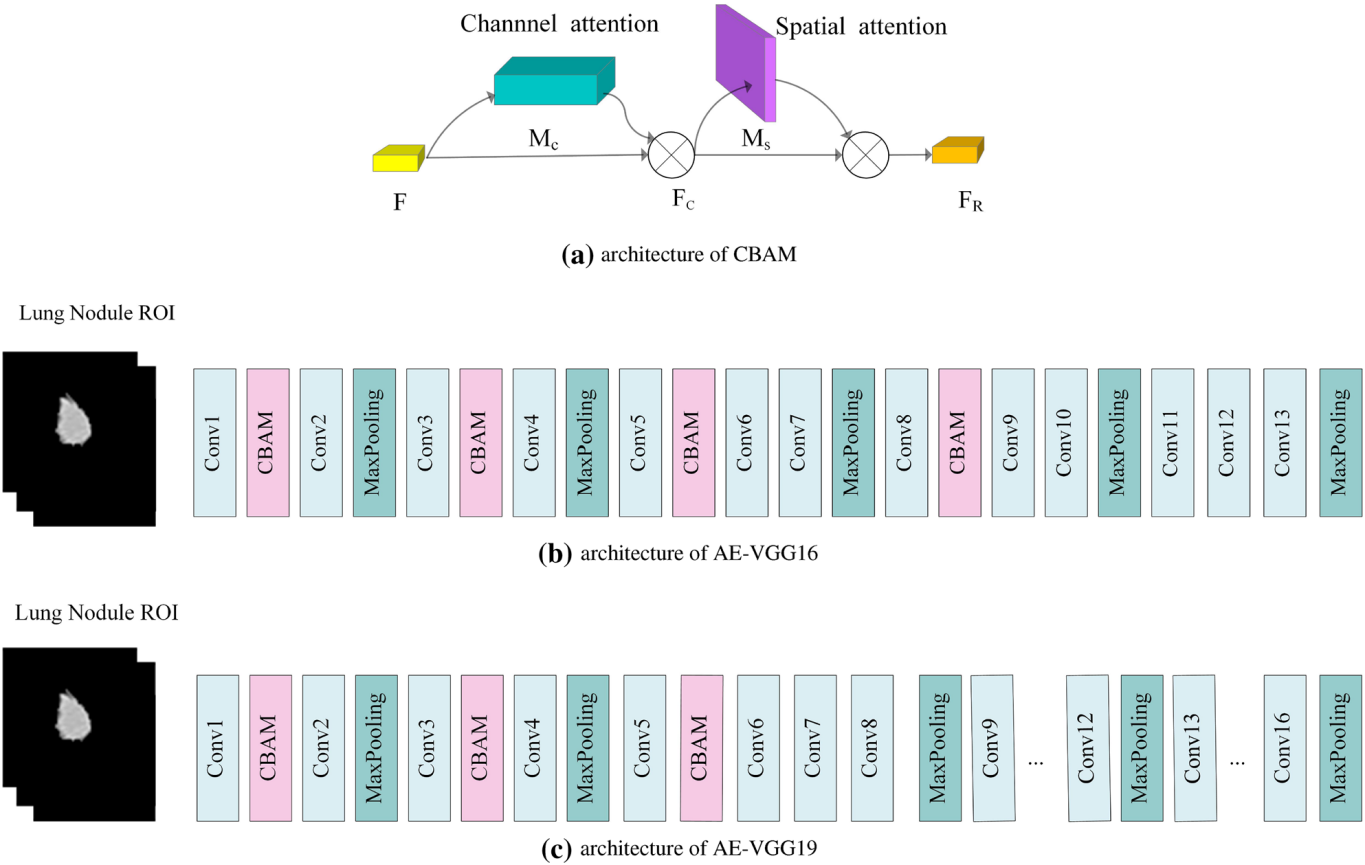
Architecture of attention-embedded VGGNet(AE-VGGNet).

So the deep feature extraction network is selected to be improved based on VGG16 and VGG19. Further, because of the ability of the attention mechanism to assign model weights, CBAM is embedded in VGG16 and VGG19, and the back-end fully connected layer is removed to construct AE-VGG16 and AE-VGG19 feature extraction models, aiming to take into account both channel and spatial information Directs the weight distribution of the model. The proposed deep feature extraction model architecture is shown in Fig. 4.

CBAM consists of a serial structure of channel attention module and spatial attention module, the architecture is shown in Fig. 4a. The network first learns what are the key features through the channel attention module, and then uses the spatial attention module to learn where the key features are, thereby strengthening the acquisition of image discriminative features. In the CBAM process, the input features of CBAM are denoted by *F*, and the output features of the channel attention mechanism and the final features output from the spatial attention mechanism are denoted as $F_C$ and $F_R$, respectively:

1 $$\begin{aligned} F_C&= M_C(F) \otimes F \end{aligned}$$

2 $$\begin{aligned} F_R&= M_S(F) \otimes F_C \end{aligned}$$

where $M_C$ represents the refined features are acquired by multiplying the input feature with the channel attention map, $M_S$ represents a sequential spatial attention map. Where $\otimes$ denotes element-wise multiplication. After the input features *F* passing through the channel attention module, the feature map $F_C$ containing more key channel information can be obtained. Then pass the $F_C$ through the spatial attention module to obtain a feature image $F_R$ containing more key information of spatial position, and use it as the final output feature map of CBAM. The channel attention module uses both average pooling and max pooling to aggregate feature map information, which helps to collect more discriminative features and infer a more effective attention channel. The spatial attention module exploits the spatial relationship between features and focuses on the location information of discriminative features, which is complementary to channel attention.

The proposed feature extraction models AE-VGG16 and AE-VGG19 are improved based on classic VGG16 and VGG19, respectively. The construction process is as follows:

1. Take the model parameters of VGG16 and VGG19 pre-trained on ImageNet as initialization parameters
2. Modify the network architecture of VGG16 and VGG19: embed CBAM after conv1, conv3, conv5, and conv8 in VGG16, and embed CBAM after conv1, conv3, and conv5 in VGG19. To be suitable for the lung nodule recognition binary classification task, the output node of the last FCL of VGG16 and VGG19 is modified to 2
3. Load the preprocessed candidate nodule ROI image, fine-tune the initialization parameters by backpropagation
4. Remove the FCL of the fine-tuned VGG16 and VGG19 models to construct feature extraction networks AE-VGG16 and AE-VGG19. The model architecture is shown in Fig. 4b and c.

The proposed feature extraction model uses a parameter-based transfer learning method to reduce the training cost, and then retrains the weights with the preprocessed candidate nodule ROI images supplement the information loss in the target domain. The low-level features of CNN have high resolution and sufficient details, but weak semantic information. Adding CBAM after the low-level convolutional layer guides the network to pay more attention to the detailed features of the nodule area from the channel and space, and improves the feature extraction ability of the network. The convolutional layer and the pooling layer are the core of feature extraction of CNN. The feature map output by the last layer of pooling layer contains the most abundant semantic information of lung nodules, which can describe the features more comprehensively. FCL plays the role of a “classifier” in the CNN network, but FCL has too many parameters and occupies a large proportion of the network, which quickly leads to the overfitting the model. In order to prevent the model from overfitting and reduce the number of parameters of the model, FCL was removed, and lung nodule recognition was realized by combining with SVM.

The deep features extracted by AE-VGG16 and AE-VGG19 are high-dimensional features with a dimension up to 25088. It contains many redundant and irrelevant features, which can easily lead to dimension disaster. Feature dimension reduction is an effective method^61^. PCA^62^ is a method of mapping high-dimensional features to low-dimensional features based on the minimum mean square error. The new feature set is an orthogonal feature set, which can preserve the information of the original data to the greatest extent. Each new feature is a linear combination of the original features, which can reflect the comprehensive information of the original features. PCA is used to reduce the dimension of the extracted deep features to reduce the calculation capacity and improve the performance of the classifier.

### Feature fusion based on CCA

The purpose of feature fusion is to combine two sets of features extracted from an image into a set of fused features more substantial information. CCA method is used to fuse the two sets of feature vectors after dimension reduction, and the characteristic information of candidate nodules is described by typical variables. Unlike the classical feature fusion method, CCA needs to first project the two sets of feature vectors into two sets of typical variables through linear changes. Then use the correlation between the two sets of typical variables to represent the overall correlation between the two sets of feature vectors.

Assume that there are two sets of feature matrices $M\in R^{p\times n}$, $N\in R^{q\times n}$. Where n is the number of samples, *p* and *q* are the dimensions of the feature vectors in *M* and *N*, respectively. The learning goal of CCA is to find the pairwise sum of projection directions $W_m\in R^p$ and $Wn\in R^q$, Make the canonical variable have the maximum correlation between $M^*=W_m^TM$ and $N^*=W_n^TN$, the objective function is the maximum correlation coefficient $\rho (M^*,N^*)$, which is defined by the correlation coefficient:

3 $$\begin{aligned} \rho (M^*,N^*)&=\frac{W_m^TS_{mn}W_n}{\sqrt{W_m^TS_{mm}W_m}\sqrt{W_n^TS_{nn}W_n}} \end{aligned}$$

Among them, $S_{mm}=MM^T$ and $S_{nn}=NN^T$ are the auto-covariance matrices of *M* and *N*, and $S_{mn}=MN^T$ is the cross-covariance matrix of *M* and *N*. The objective function is transformed into:

4 $$\begin{aligned} \begin{aligned}{}&max\ w_m^TS_{mn}w_n \\&\quad st.\ w_m^TS_{mn}w_m=1, w_n^TS_{nn}w_n=1 \end{aligned} \end{aligned}$$

In this way, the first pair of canonical variables with the largest correlation between the two groups of variables can be obtained, and the correlation coefficient between them is called the correlation coefficient of the first pair of canonical variables. Then continue to construct the second pair of canonical variables according to this method; By analogy, all *k* pairs of canonical variables can be obtained to form two sets of canonical variables $X^*$ and the value range of $\rho$ is [0,1]. The closer $\rho$ is to 1, the greater the correlation between the two sets of features. The top-ranked canonical variables have higher correlation and better feature representation for the original image.

The typical variables extracted by CCA can be fused according to the classic feature fusion method, which are the serial fusion method represented by $Z_{con}$ and the parallel fusion method represented by $Z_{sum}$, as shown in Eqs. (5) and (6):

5 $$\begin{aligned}{}&Z_{con}=\left[ \begin{array}{c} W_m^TM \\ W_n^TN \end{array} \right] =\left[ \begin{array}{c} M^* \\ N^* \end{array} \right] \end{aligned}$$

6 $$\begin{aligned}{}&Z_{sum}=W_m^TM+W_n^TN=M^*+N^* \end{aligned}$$

For the same image, although there are differences in the features extracted by different architectures, the feature vectors with more significant correlation between different feature groups describe more discriminative vital features. According to the correlation coefficient $\rho$, the first m pairs of typical variables are selected. Then the $l_1(\le l)$ pairs of typical variables are fused by serial or parallel fusion, and the optimized fusion features are used as the input of the next stage classifier.

### Lung nodule recognition based on MKL-SVM-IPSO algorithm

With its theoretical foundation and generalization ability, SVM has become a powerful tool for solving binary classification problems^63–65^. The MKL-SVM formed by combining kernel functions with different properties will have the properties of different kernel functions, which can improve the classification accuracy and robustness^66^. In order to obtain better learning ability and generalization ability, the RBF kernel function and the polynomial kernel function are combined into a multi-kernel function in the form of linear convex combination, and the MKL-SVM is the same as the literature^22^. The polynomial kernel function, radial basis kernel function and multi-kernel function are denoted $K_{poly}$, $K_{rbf}$ and $K_{mix}$, respectively:

7 $$\begin{aligned}{}&K_{poly}(x,y)=(x^ty+1)^d \end{aligned}$$

8 $$\begin{aligned}{}&k_{rfb}(x,y)=exp(-\Vert x-y\Vert ^2/2g^2) \end{aligned}$$

9 $$\begin{aligned}{}&K_{mix}(x,x')=\gamma K_{poly}(x,x')+(1-\gamma )K_{rbf}(x,x') ,0<\gamma <1 \end{aligned}$$

Among them, the parameter d represents the order of the polynomial kernel function, which is a positive integer. The parameter g represents the kernel width of the RBF kernel.$\gamma$ represents the proportion of the two kernel functions in the multi-kernel function.When the grid search algorithm is used, although the optimal global solution can be found, the MKL-SVM model contains many parameters and requires multiple layers of nested loops, which results in a large amount of computation and a long training time. The PSO imitates the foraging of birds, and it is more purposeful to find the optimal target through the information sharing of the birds, and can find the optimal solution in a shorter time^67^. An improved Particle Swarm Optimization(IPSO) is proposed for parameter optimization of the MKL-SVM model to speed up the training speed and shorten the training time of the model.

The PSO algorithm treats the potential solutions of the model as particles in the search space. Assuming that in a D-dimensional target search space, there is a particle swarm composed of n particles $X=(X_1,X_2,\cdots ,X_n)$, the position and velocity of the i-th particle are $X_i=(x_{i1},x_{i2},\cdots ,x_{iD})^T$ and $V_i=(V_{i1},V_{i2},\cdots ,V_{iD})^T$ respectively, and the optimal solution found in the i-th particle is the individual extremum expressed as $P_i=(P_{i1},P_{i2},\cdots ,P_{iD})^T$, all particles The overall optimal solution found is the global extremum denoted as $P_g=(P_{g1},P_{g2},\cdots ,P_{gD})^T$. The particle updates the velocity and position of each generation through the individual extremum and the group extremum, which are expressed as follows:

10 $$\begin{aligned} V_{id}^{k+1}&=\omega V_{id}^k+c_1r_1(P_{id}^k-X_{id}^k)+c_2r_2(P_{gd}^k-X_{id}^k) \end{aligned}$$

11 $$\begin{aligned} X_{id}^{k+1}&=X_{id}^K+V_{id}^{k+1} \end{aligned}$$

In the above formula, $c_1$ and $c_2$ are the learning factor, which is a non-negative constant; $r_1$ and $r_2$ are random numbers distributed in the [0,1] interval; $d=1,2,\cdots , D$, where *D* represents the number of parameters to be searched *k* is the current number of iterations, $X_{id}^k$ and $V_{id}^k$ respectively represent the position and velocity of particle *i* on the $d-th$ parameter in the $k-th$ iteration; $P_{id}^k$ and $P_{gd}^k$ respectively represent the individual extremum and the global extremum on the parameter. In order to prevent the blind search of particles, their position and velocity are usually limited within the interval [-$X_{max}$,$X_{max}$] and [-$V_{max}$,$V_{max}$] interval according to experience.

In the PSO algorithm, inertial weights $\omega$ reflect the ability of particles to inherit previous velocities. A larger inertia weight value is more favourable for global search, and a smaller weight value is more favourable for local search^68^. The adaptive inertia weight supervises the current position and velocity of the particles in the search space, calculates the fitness value of the particle, and dynamically adjusts the inertia weight through the feedback fitness value, avoiding the premature maturity of the particle and helping to obtain the optimal global solution. Therefore, an adaptive inertia weight strategy of hierarchical adjustment is formulated, and the population is divided into two types of subgroups according to the fitness value of the particles. The corresponding weight adjustment strategy is adopted for the particles in different subgroups. The proposed adaptive inertia weight strategy is as follows:

12 $$\begin{aligned} \omega ={\left\{ \begin{array}{ll} \omega _s-\frac{(f_i-f_{max})\times (\omega _s-\omega _e)}{f_{avg}-f_{max}}, &{}f_i\le f_{avg} \\ \omega _e, &{}f_i \&{}gt;f_{avg} \end{array}\right. } \end{aligned}$$

In formula (12), $\omega$ represents the inertia weight; represents the initial inertia weight, $\omega _s$ represents the maximum number of iterations, $f_i$ is the current fitness value of the i-th particle, and $f_{avg}$ and $f_{max}$ are the average and maximum current fitness value of all particles, respectively. The specific steps of the proposed adaptive inertia weight strategy are as follows: calculate the average value $f_{avg}$ of the fitness of all the particles at present, take the particles whose fitness value is greater than $f_{avg}$ to be divided into the same subgroup, and set the inertia weight value at the current moment as the initial inertia weight. The remaining particles meet the condition that the fitness value is less than or equal to $\omega _s$. The remaining particles are divided into another subgroup. The adaptive inertia value of the current particle is calculated according to the formula under the corresponding condition of formula (12).

Further, the learning factor $c_1$ and $c_2$ determines the influence of the particle’s own experience and the group’s experience on the particle’s trajectory, and setting a larger or smaller value is not conducive to particle search^69^. $c_1$ is the self-learning factor, which means the influence weight of the optimal position experienced by the particle on the particle action. $c_2$ is the social learning factor, which represents the influence weight of the optimal position of the particle group on the particle action. The improved $c_1$ and $c_2$ respectively are shown in formula (13) and (14):

13 $$\begin{aligned} c_1&=c_{max}-\frac{(t-1)}{T}\times c_{max} \end{aligned}$$

14 $$\begin{aligned} c_2&=c_{min}+\frac{(t-1)}{T}\times c_{min} \end{aligned}$$

It can be seen from the above formula that in the optimization process, the particles in the initial stage have strong self-learning ability and weak collective learning ability. As the number of iterations increases, the dynamic learning factor $c_1$ changes from large to small, and $c_2$ from small to large, the joint learning ability of particles is strong. Still, the self-learning ability is weak, which helps to obtain the optimal global solution and avoid falling into the local optimal solution.

**Figure 5:**
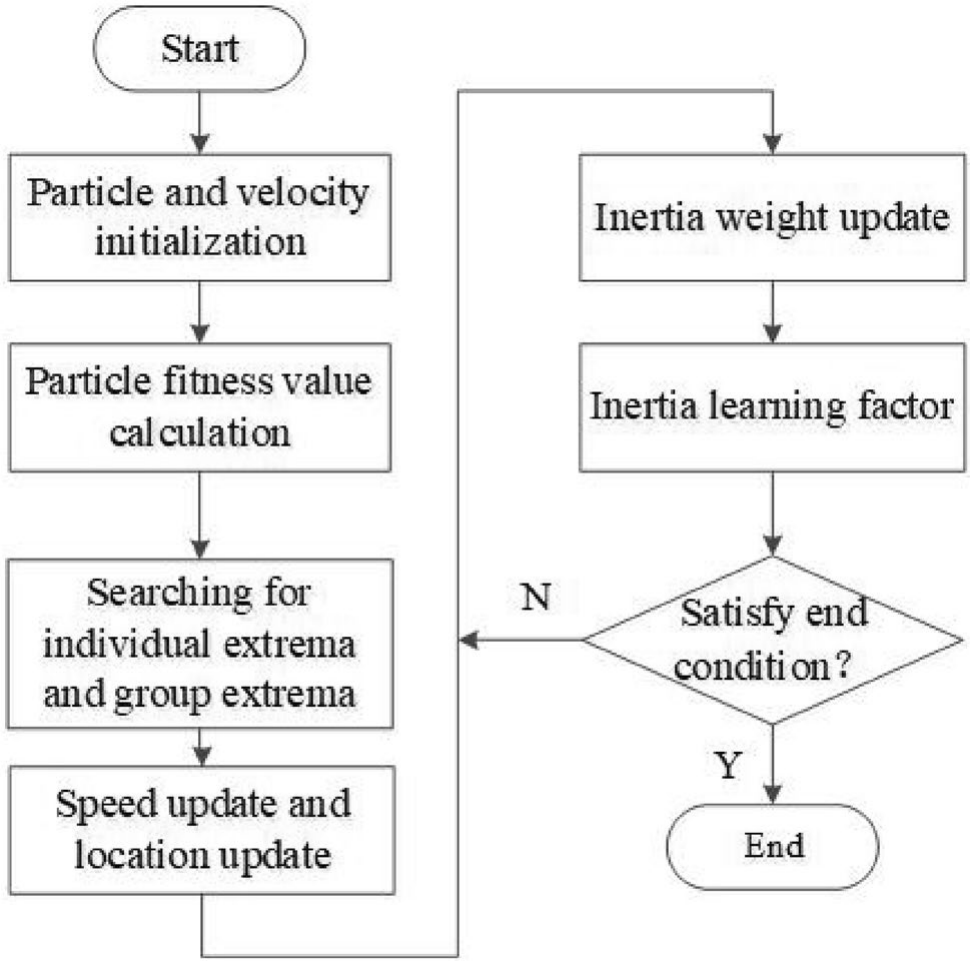
The flowchart of the MKL-SVM-IPSO algorithm.

The proposed MKL-SVM-IPSO algorithm was used for lung nodule recognition. Firstly, in order to obtain better learning ability and generalization ability, MKL-SVM is constructed with polynomial kernel and RBF kernel. Then, to speed up the parameter optimization process, the PSO algorithm with adaptive inertia weight is introduced into the MKL-SVM, which can adaptively optimize the parameters according to the fitness value and speed up the model’s training. At the same time, dynamic learning factors are introduced to adjust the self-learning ability and collective learning ability of particles. The proposed algorithm solves the problem of slow model training and makes it easy to fall into local optimum. The flowchart of the MKL-SVM-IPSO algorithm is shown in Fig. 5.

The above steps are as follows:

1. Initialize the position and velocity of particles;
2. Calculate the fitness value of each particle, and store the current status and fitness value of each particle;
3. Find out the individual extremum and the global extremum with the best fitness value in the present particle;
4. Update the speed and position of the particle;
5. Calculate the fitness value of the particle according to the updated speed and position, and then dynamically update inertia weight through the feedback fitness value;
6. Adjust the self-learning ability and collective learning ability of particles through the inertial learning factor;
7. Check whether the individual extremum and the group extremum meet the termination conditions. If satisfied, stop the calculation and get the optimal parameter group; if not, continue to repeat step (5).

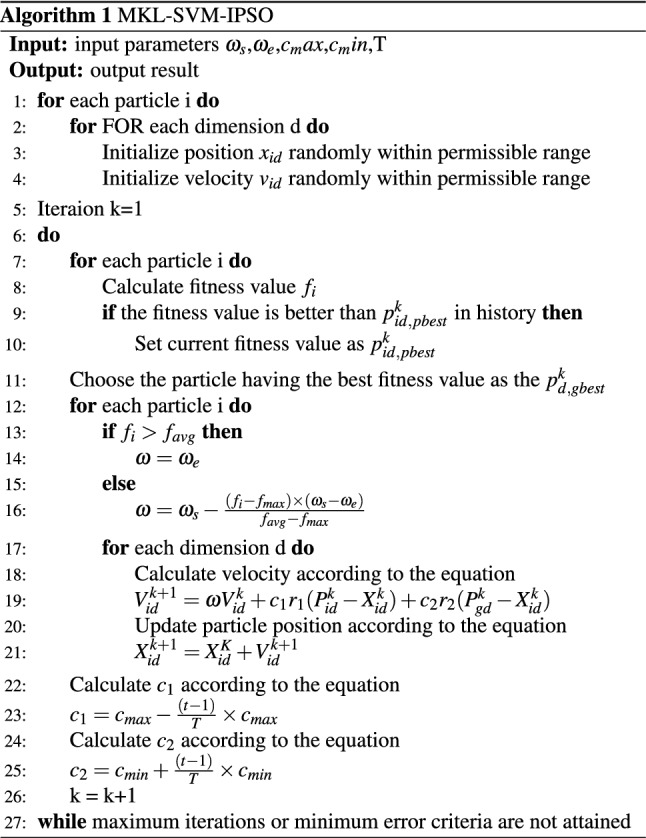

## Experimental results and analysis

### Experimental parameter settings

During the training phase of the AE-VGG16 and AE-VGG19 feature extraction models, the pre-trained weights are fine-tuned using a stochastic gradient descent (SGD) method. Based on experience and taking into account the hardware conditions of the laboratory, the momentum factor c is set to 0.9, the initial learning rate is set to 0.001, the batch size is set to 32, and the number of iterations is 50. To match the standardized input size of the pre-trained model, it is necessary to reconstruct the preprocessed lung nodule ROI image size to 224224, and then input the feature extraction models AE-VGG16 and AE-VGG19 to extract 77512=25088 dimensional features, and finally reduce the dimensionality of the two sets of feature sets through PCA.

In the feature fusion stage using CCA, the corresponding feature vectors are selected respectively according to the value range of the correlation coefficient. Then, the fusion feature set is composed of serial or parallel fusion strategy, and input into the MKL-SVM-IPSO recognition algorithm. Finally, compare the recognition results and select the optimal fusion feature as the input of the recognition algorithm in the next stage. The value range of the correlation coefficient $\rho$ is [0.6, 1].

In the parameter optimization stage of the MKL-SVM-IPSO identification algorithm. The particle swarm position and velocity are initialized. The training uses 5-fold cross-validation. The particle swarm size is set to $n=20$, and the size of each particle is $D=3$, corresponding to the regularization coefficient *C* to be searched, the RBF kernel width *g*, and the weight $\gamma$ of the multi-kernel function. Among them, the selection range of the regularization coefficient *C* is between $2^{-9}$ and $2^{9}$, the selection range of the kernel width g of the RBF kernel function is between $2^{-7}$ and $2^{7}$, and $\gamma$ represents the proportion of the two kernel functions in the multi-kernel function. The range is selected between 0 and 1.

The parameters of the PSO, inertia weight $\omega _s=0.9$, $\omega _e=0.4$, learning factors $c_1=2$, $c_2=2$, $c_{1max}=2.5$, $c_{2max}=2.5$, $c_{1min}=0.5$, $c_{2min}=0.5$ are selected according to experience, so that the inertia weight decreases linearly from the initial 0.9 to 0.4. When the order d of the polynomial kernel function is small, the generalization ability is strong. In order to reflect the nonlinear characteristics, *d* is selected as 3. The weight value in the multi-kernel function is a very important parameter, which directly affects the components occupied by each basic kernel function in the multi-kernel function. The weight coefficient *m* is selected as [0, 1], and the maximum number of iterations of the experiment is set to 200.

### Evaluating indicator

The evaluation indicators of the experiment mainly used accuracy (ACC), sensitivity (SEN), F1-score and Receiver operating characteristic (ROC) curve. The expressions are shown in Eqs. (15), (16) and (18):

15 $$\begin{aligned} ACC&=\frac{TP+TN}{TP+TN+FP+FN} \end{aligned}$$

16 $$\begin{aligned} SEN&=\frac{TP}{TP+FN} \end{aligned}$$

17 $$\begin{aligned} PRE&=\frac{TP}{TP+FP} \end{aligned}$$

18 $$\begin{aligned} F1-score&=\frac{2*SEN*PRE}{SEN+PRE} \end{aligned}$$

Among them, TP is the identified true positive nodule; TN is true negative; FP is false-positive, and FN is false-negative.

ACC represents the correct rate of overall recognition. SEN stands for the proportion of correctly identified nodules to all nodules, also known as recall, which reflects the ability to identify positive samples. PRE represents the proportion of the number of correctly identified nodules to the number of nodules identified as the result, reflecting the ability to distinguish negative samples. F1-score and ROC curve were further used as comprehensive evaluation indicators. F1-score is the weighted harmonic mean of PRE and SEN, the higher the value, the more robust the recognition model. The horizontal axis of the ROC curve is specificity, and the vertical axis is sensitivity, which is used to evaluate the predictive ability of the model.

### Experimental result analysis

In order to evaluate the effectiveness of the key algorithms of the proposed lung CAD system, the experiment is divided into four parts.

The first part is the ablation experiment of the feature extraction network, which aims to verify the effectiveness of the proposed AE-VGG16 and AE-VGG19 feature extraction networks. The second part is the feature fusion experiment. According to the correlation coefficient, the features of different dimensions are selected. Combined with the serial or parallel feature fusion strategy, the fusion feature with the best recognition result is chosen as the input of the following stage recognition algorithm. The third part is the recognition algorithm experiment, including the comparison experiment before and after the improvement of the algorithm, and the comparison experiment with other different classifier algorithms to verify the validity of the MKL-SVM-IPSO recognition algorithm. The fourth section is a comparative experiment with the baseline algorithms of other lung CAD systems to demonstrate the competitiveness of the proposed lung CAD system. To ensure the robustness of the experimental results, each group of experiments was repeated 5 times, and the average of the experimental results was taken as the final experimental result.

#### Feature extraction network ablation experiments

First, in order to verify that VGGNets are more suitable for the research task, the more popular CNN architectures: ResNet18, ResNet34, ResNet50, DenseNet121 and MobilNet V2 are selected for comparative experiments. The results are shown in Table 1.

**Table 1:** Comparative experiment of different architectures.

Network	Dimension	ACC (%)	SEN (%)	SEN (%)
ResNet18	98	91.32	97.16	0.9326
ResNet34	98	92.98	95.28	0.938
ResNet50	98	85.75	94.81	0.8874
DenseNet121	98	91.93	95.64	0.9326
MobilNet V2	98	87.02	89.08	0.8946
VGG16	98	**97.81**	**97.69**	**0.982**
VGG19	98	**96.4**	**94.85**	**0.9683**

Significant values are in [bold]

By analyzing the data in Table 1, we can find that VGG16 and VGG19 achieve better performance compared to ResNet18, ResNet34, ResNet50, DenseNet121 and MobilNet V2. Therefore, VGG16 and VGG19 are selected as the basic models of the feature extraction network.

As discussed above, through the AE-VGG16 and AE-VGG19 feature extraction models,25088 dimensional features were extracted ,respectively. On this basis, PCA was used for feature dimension reduction. Table 2 lists the cumulative variance contribution rates corresponding to different feature dimensions selected by the four feature extraction models. It is the proportion of the original data information carried by the selected principal components after dimension reduction.

**Table 2:** The cumulative variance contribution rate corresponding to different feature dimensions selected by the feature extraction model.

Model	Dimension					
	49 (%)	98 (%)	147 (%)	196 (%)	245 (%)	294 (%)
VGG16	82.467	91.188	95.055	97.077	98.207	98.917
AE-VGG16	99.653	99.925	99.954	99.991	99.996	99.998
VGG19	84.328	92.718	96.131	97.853	98.771	99.285
AE-VGG19	99.653	99.925	99.977	99.991	99.996	99.998

Significant values are in [bold]

It can be seen from Table 2 that the cumulative variance contribution rate of the first 98-dimensional features of the proposed AE-VGG16 and AE-VGG19 are both 99.925%, so retaining the first 98-dimensional features can represent almost all the information of the original data. The cumulative variance contribution rates of the first 98-dimensional features of the VGG16 and VGG19 feature dimensions are 91.188% and 92.718%, respectively, and the cumulative variance contribution rates of the first 294-dimensional features in the feature dimension interval can reach 98.917% and 99.285%. The experimental results show that compared with VGG-16 and VGG-19, the proposed AE-VGG16 and AE-VGG19 can still improve the feature expression ability of the network after PCA dimensionality reduction, and make the network pay more attention to the key information of nodules, and can retain the information of the original data to a greater extent with lower-dimensional features.

To verify the effectiveness of PCA dimensionality reduction, we compared the experimental results of reducing the depth features of AE-VGG16 and AE-VGG16 from the original 25088 dimensions to 98, 196 and 294 dimensions, respectively. The results are shown in Table 3.

**Table 3:** Recognition results of reducing deep features to different dimensions using PCA.

Network	Dimension	ACC (%)	SEN (%)	SEN (%)
AE-VGG16	98	**98.25**	**97.99**	**0.9845**
	196	98.95	98.41	0.9904
	294	97.11	98.5	0.9731
AE-VGG19	98	**99.39**	**99.84**	**0.9945**
	196	98.51	98.95	0.9873
	294	95	98.79	0.9584

Significant values are in bold.

By analyzing the data in Table 3, we can find that reducing AE-VGG16 to 196 dimensions achieves better results than reducing to 98 and 294 dimensions. In order to reduce the amount of computation without sacrificing too much accuracy, the 98-dimensional feature of AE-VGG16 is selected. Optimal results were obtained when AE-VGG19 was reduced to 98 dimensions, so the 98-dimensional features of AE-VGG19 were chosen.

Further, in order to verify whether the embedded attention mechanism can enhance the expressive ability of the feature extraction network, the original VGG network and the AE-VGG network were compared as the feature extractor respectively and the recognition results were carried out using the MKL-SVM-IPSO recognition algorithm. The results are listed in Table 4.

**Table 4:** The cumulative variance contribution rate corresponding to different feature dimensions selected by the feature extraction mode.

Model	CBAM	Dimension	ACC (%)	SEN (%)	F1-score
VGG16		98	97.81	97.69	0.9820
AE-VGG16	$\surd$	98	98.25	97.99	0.9845
VGG19		98	96.4	94.85	0.9683
AE-VGG19	$\surd$	98	99.39	99.84	0.9945

Significant values are in bold.

It can be seen from Table 4 that the ACC SEN F1-score indicators of the AE-VGG16 and AE-VGG19 networks with the introduction of the attention mechanism are better than the original networks VGG16 and VGG19. The ACC, SEN, and F1-score of AE-VGG16 reached 98.25%, 97.99%, and 0.9845, respectively, slightly improved compared with the original VGG16. The ACC, SEN, and F1-score of AE-VGG19 reached 99.39%, 99.84%, and 0.9945, respectively. Compared with the original VGG19, the three indicators of AE-VGG19 were improved by 2.99%, 4.99%, and 0.0262, respectively. The above experimental results verify that embedding the attention mechanism into the feature extraction network can improve the feature expression ability.

Further, other attention modules SE-Net and SK-Net are embedded in the same position of VGG16, and the method proposed in this paper is used to build a feature extraction model. The feature vector after PCA dimension reduction is used as input. The recognition results on the test set with different attention modules embedded are listed in Table 5.

**Table 5:** Recognition results with different attention modules embedded.

Network	Module	Dimension	ACC (%)	SEN (%)	F1-score
VGG16		98	96.37	94.71	0.9686
VGG16	SE-Net	98	97.77	97.33	0.9788
VGG16	SK-Net	98	97.81	97.69	0.982
VGG16	CBAM	**98**	**98.25**	**97.99**	**0.9845**

Significant values are in bold.

It can be seen from Table 5 that the networks embedded with the attention module are better than the original network VGG16. Among them, the performance of the model embedded in CBAM is the best, followed by SK-Net and worse by SE-Net. In summary, the experiments in this paper will use the AE-VGG16 and AE-VGG19 networks to extract 98-dimensional feature vectors, respectively.

**Figure 6:**
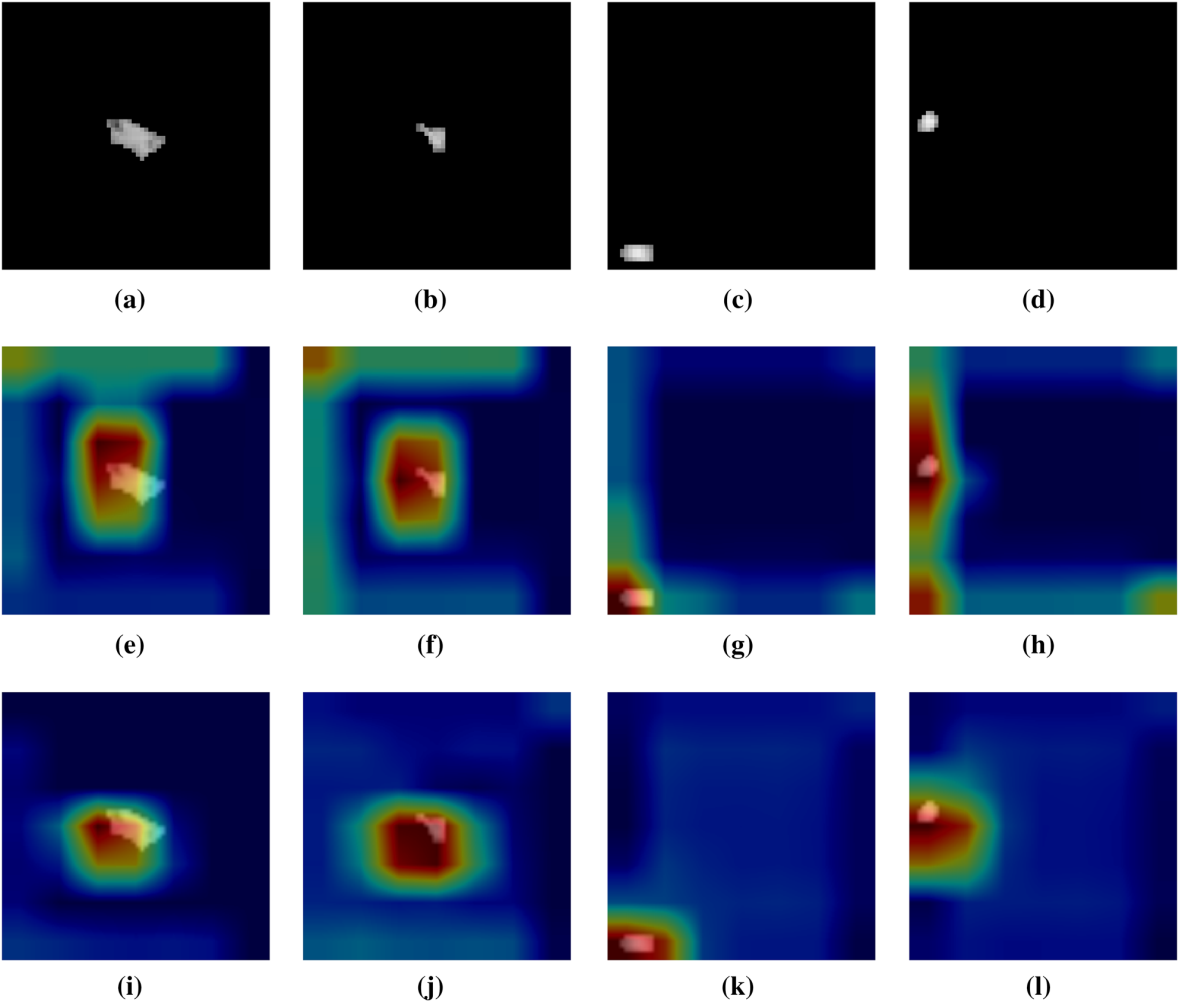
The Grad-CAM maps. (**a**)–(**d**) Preprocessed sample image. (**e**)–(**h**) Corresponding AE-VGG16 image. (**i**)–(**l**) Corresponding AE-VGG19 image.

To determine which features were learned by the proposed AE-VGG16 and AE-VGG19 networks and which regions in the input lung nodule ROI images were activated. We use gradient-weighted class activation mapping (Grad-CAM) to extract gradients from AE-VGG16 and AE-VGG19 respectively and highlight the most important regions^70^. The Grad-CAM maps corresponding to the preprocessed sample image is shown in Fig. 6 .

In general, the red areas on the Grad-CAM maps represent the areas that have received the most attention in the network, while the blue areas are the areas that have received the least attention. By looking at Fig. 6, it was found that both AE-VGG16 and AE-VGG19 could almost focus on the nodular region. If the more related features between the two are fused, the performance of the model can be further improved.

#### Feature fusion experiment

In the experiment, CCA is selected as the feature fusion algorithm, and the corresponding feature vector is determined according to the value range of the correlation coefficient. And choose serial or parallel fusion strategies to compose fusion features, respectively. Table 6 shows the experimental results when different fusion methods and correlation coefficients $\rho$ are determined. The serial and parallel fusion methods are denoted as “concat” and “sum”, respectively.

**Table 6:** Experimental results of selecting different feature fusion methods.

Model	Fusion method	$\rho$	Dimension	ACC (%)	SEN (%)	F1-score
AE-VGG16			98	98.25	97.99	0.9845
AE-VGG19			98	99.39	99.84	0.9945
AE-VGG16 + AE-VGG19	con	$\rho \ge$0.9	84	99.12	99.04	0.9927
		$\rho \ge$**0.8**	**118**	**99.56**	**99.30**	**0.9965**
		$\rho \ge$0.7	138	98.77	98.91	0.9891
		$\rho \ge$0.6	152	97.98	96.94	0.9812
AE-VGG16 + AE-VGG19	sum	$\rho \ge$0.9	42	97.72	96.09	0.9800
		$\rho \ge$**0.8**	**59**	**99.21**	**99.13**	**0.9930**
		$\rho \ge$0.7	69	98.68	98.43	0.9882
		$\rho \ge$0.6	76	98.25	97.83	0.9854

Significant values are in bold.

It can be seen from Table 6 that under the two fusion strategies, the fusion feature results when the correlation coefficient $\rho >0.8$ is relatively better, so the subsequent experiments will use the fusion features corresponding to $\rho >0.8$. When using the serial fusion strategy, compared with AE-VGG16 without feature fusion, the ACC and SEN are improved by 1.31%, and the F1-score is improved by 0.012; compared with AE-VGG19 without feature fusion. Compared with that, the ACC has increased by 0.17%, and the F1-score has increased by 0.002. When using the parallel fusion strategy, compared with AE-VGG16 without feature fusion, the ACC, SEN and F1-score are improved by 0.96%, 1.14% and 0.0085, respectively. When the correlation coefficient $\rho >0.8$, compared with the parallel fusion strategy, the fusion features composed of the serial fusion strategy obtained better recognition results, the ACC and SEN were increased by 0.35% and 0.17%, respectively, and the F1-score was improved. 0.0035. Although there is no significant difference in performance between our proposed method and only AE-VGG19, the ACC increases by 0.17%, and the F1-score also increases by 0.2%, considering that our final proposed model is more robust.

Therefore, the first 59-dimensional features with a correlation coefficient $\rho >0.8$ are selected. The fusion features obtained by the serial fusion strategy are used as the input of the next-stage recognition algorithm.

In addition, we performed an experiment of directly using CCA for fusion to verify the effectiveness of the proposed method. The features of AE-VGG16 and AE-VGG19 do not use PCA for dimensionality reduction, but directly use CCA for feature fusion, and the experimental results obtained are shown in Table 7.

**Table 7:** Experimental results of direct feature fusion.

Model	Fusion method	$\rho$	Dimension	ACC (%)	SEN (%)	F1-score
AE-VGG16 +AE-VGG19	concat	$\rho \ge$0.9	156	95.79	97.78	0.9649
		$\rho \ge$0.8	198	96.49	97.29	0.9649
AE-VGG16 +AE-VGG19	sum	$\rho \ge$0.9	79	97.02	98.99	0.9759
		$\rho \ge$**0.8**	99	96.45	96.19	0.9704

Significant values are in bold.

By comparing the experimental results in Table 6 and Table 7, we found that compared with the method that directly uses CCA for feature fusion, the method of dimensionality reduction and fusion can achieve better results. Although direct use of CCA for feature fusion can fuse the more relevant features between AE-VGG16 and AE-VGG19, it will lose part of the feature information, thereby reducing the recognition results. At the same time, the dimensionality reduction and fusion method proposed by us finally obtains a lower dimensionality of fusion features, which can reduce the computational load of the classifier in the next stage.

#### Recognition algorithm experiment

**Figure 7:**
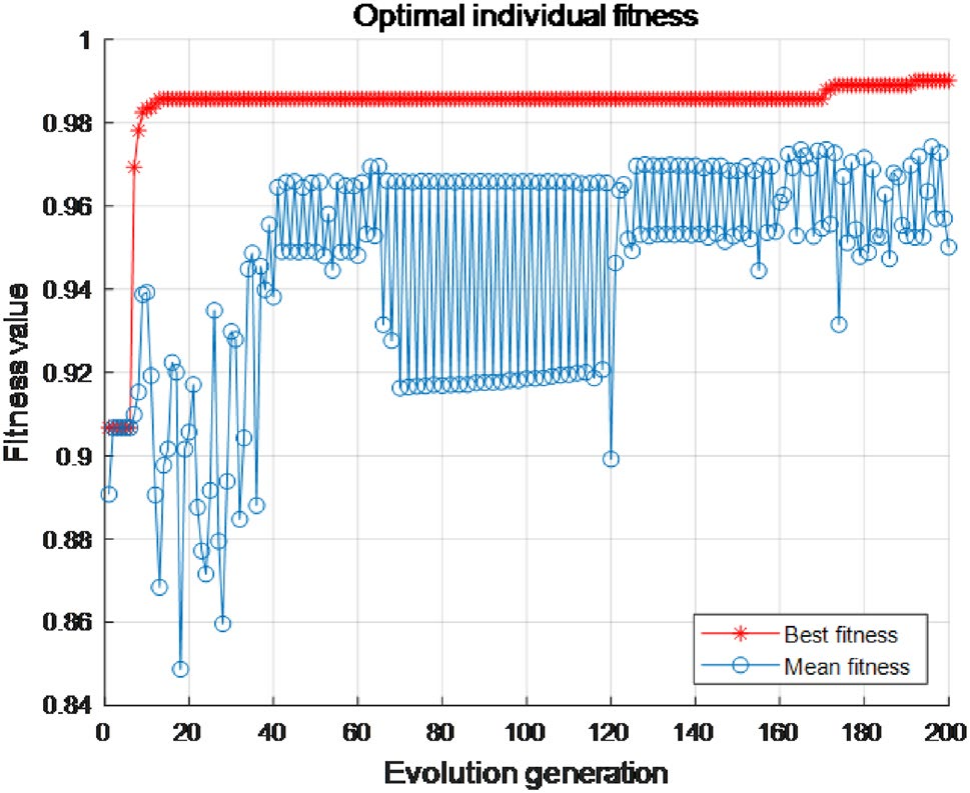
The fitness curve of the proposed algorithm.

In order to verify the effectiveness of the proposed MKL-SVM-IPSO recognition algorithm, it is compared with the recognition results of the SVM using the PSO algorithm for a single RBF kernel and the MKL-SVM using the PSO algorithm to combine the RBF and the polynomial kernel function in a convex manner. For comparison, the results are shown in Table 8. The ROC curves of the proposed MKL-SVM-IPSO algorithm in the training phase compared with other algorithms are further demonstrated in Fig. 7. AUC (Area Under the ROC) in Table 8 is the area under the ROC curve, and the larger the value, the higher the recognition effect.

**Table 8:** Results of different recognition algorithms.

Kernel Function	PSO	IPSO	ACC (%)	SEN (%)	F1-score	AUC
RBF	$\surd$		94.82	95.37	0.9567	0.9802
RBF+ poly	$\surd$		98.68	99.04	0.9868	0.9981
RBF+ poly		$\surd$	**99.56**	**99.30**	**0.9965**	**0.9989**

Significant values are in bold.

It can be seen from Table 8 that the proposed MKL-SVM-IPSO has the most substantial parameter optimization ability, and the RBF-SVM-PSO algorithm has the lowest recognition result. The AUC of the proposed MKL-SVM-IPSO recognition algorithm is the best at 0.9989; MKL-SVM-PSO is the second, and AUC is 0.9981. Figure 7 shows the fitness curve of the training phase of the proposed MKL-SVM-IPSO recognition algorithm by selecting the serial fusion strategy and the corresponding first 59-dimensional features when the correlation coefficient is $\rho >0.8$ as the input.

**Figure 8:**
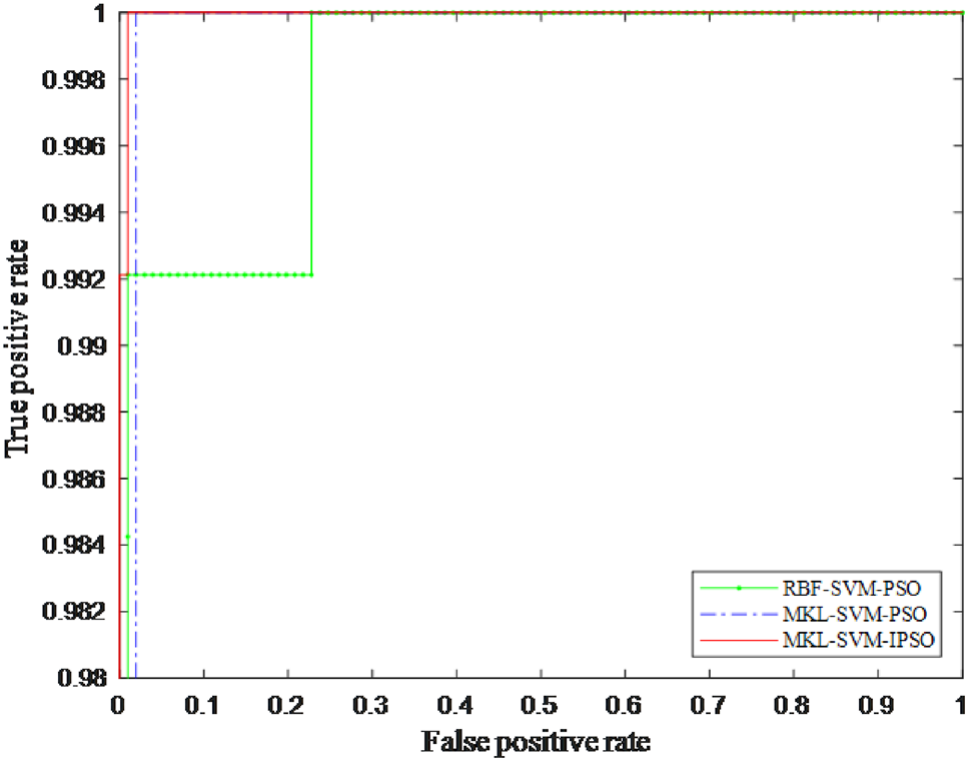
ROC curves of different recognition algorithms.

As shown in Fig. 7, the proposed MKL-SVM-IPSO has excellent parameter optimization ability, and the optimal fitness value can reach 0.9956. Although the optimal fitness value of the particle in the early search process has always been lower than 0.92, it can quickly jump out of the local optimal solution after only 20 iterations, and the optimal fitness value quickly rises above 0.98. And continue to search for better values, and finally obtain the optimal global solution. The ROC curves of the proposed MKL-SVM-IPSO algorithm in the training phase compared with other algorithms are further shown in Fig. 8.

As shown in Fig. 8, the AUC value of the proposed algorithm can reach 0.9989. The above experimental results show that the proposed MKL-SVM-IPSO can improve the convergence accuracy of the particles, thereby improving the recognition performance of lung nodules.

To further verify the effectiveness of the proposed MKL-SVM-IPSO, it is compared with the classical classifiers KNN, RF, FCL with softmax, and AdaBoost, respectively. The results are shown in Table 9.

**Table 9:** Recognition performance of different classifiers.

Classifiter	ACC (%)	SEN (%)	F1-score (%)
Softmax	96.740.3	98.650.3	97.190.5
RF	96.330.4	96.900.5	96.50.6
KNN	93.73	90.84	91.82
Adaboost	97.84	98.06	97.94
MKL-SVM-IPSO	**99.560.2**	**99.300.3**	**99.650.2**

Significant values are in bold.

It can be seen from Table 9 that the proposed MKL-SVM-IPSO has the best recognition performance. To sum up, the values of all evaluation indicators are above 90%, and the standard deviation of performance indicators is below 0.6%, indicating that the extracted fusion features can fully describe the critical information of lung nodules. At the same time, by analyzing the recognition performance of different classifiers combined with fusion features, it is verified that the proposed MKL-SVM-IPSO recognition algorithm has better performance.

#### Comparison experiments with baseline algorithms of other lung CAD systems

In order to further evaluate the effectiveness of the proposed key algorithm in the lung CAD system, some experiments performed on the LUNA16 dataset were selected for comparison, and the results are listed in Table 10.

**Table 10:** Recognition performance of different classifiers.

References	Year	Datasets	Methods	ACC(%)	SEN(%)
Shi et al.^37^	2019	LUNA16(1400 images)	Fine-tuning VGG16 features + SVM	87.8	87.2
Mastouri et al.^38^	2020	LUNA16 (3186 images)	Two-stream CNNs (VGG16 and VGG19) + SVM	91.99	91.85
Chang et al.^55^	2021	LUNA16(1140 images)	handcrafted features + VGG16 + Cascade + Hybrid Swarm Intelligence Optimization for MKL-SVM	95.88	91.97
proposed	2022	LUNA16(1140 images)	AE-VGG16+AE-VGG19 + CCA+ MKL-SVM-IPSO	**99.56**	**99.30**

Significant values are in bold.

It can be seen from Table 10 that the literature^37^ uses the fine-tuned VGG16 model to extract deep features, and combines with the input SVM to identify whether the candidate ROI is a nodule. Reference^38^ used the bilinear CNN model composed of VGG16 and VGG19 as the feature extractor, and input SVM to realize lung nodule recognition. Reference^55^ concatenates handcrafted features with deep features extracted by VGG16 model, and uses a Simulated Annealing algorithm combined with PSO to identify lung nodules. Compared with the above methods, the proposed lung CAD system achieves better recognition results, has certain competitiveness, can effectively avoid the occurrence of false detection and missed detection, and improve the recognition accuracy of lung nodules.

**Figure 9:**
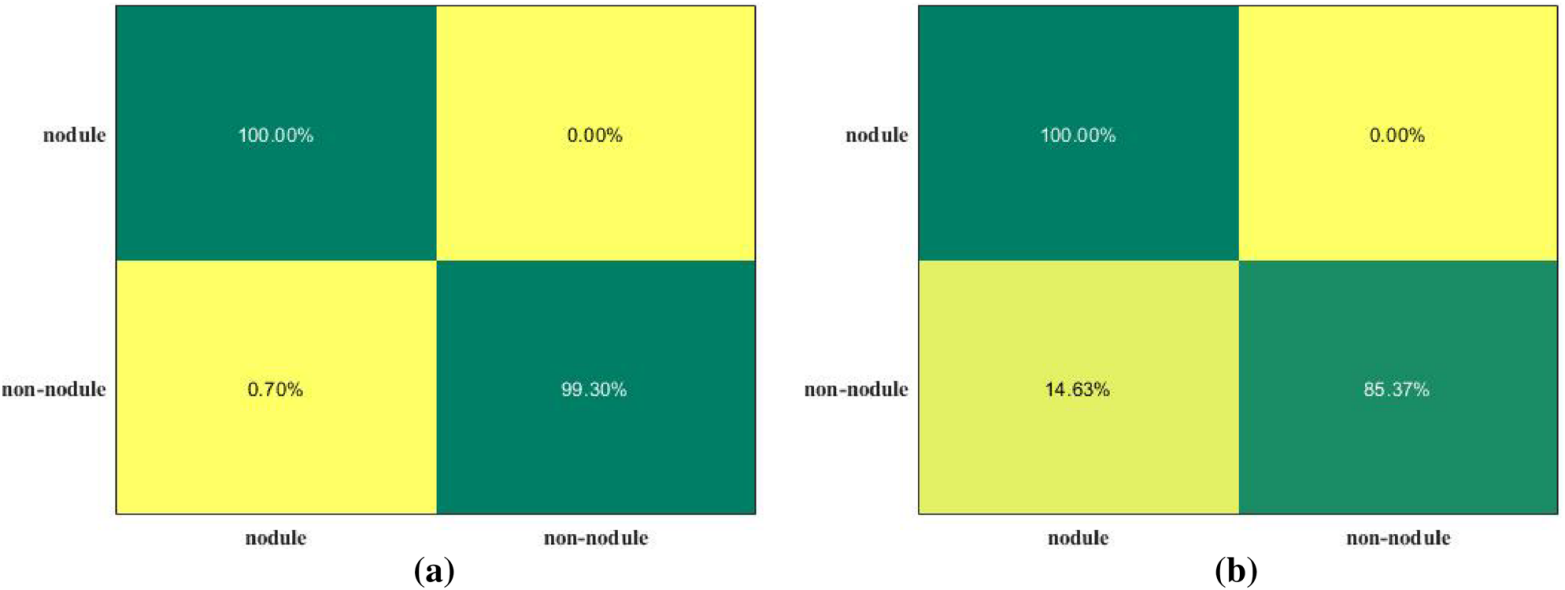
The confusion matrix of the proposed lung CAD system.

To verify the effectiveness and robustness of the proposed model, in addition to the LUNA16 experimental dataset, the public dataset DeepLesion^53^was added as a test set to verify the generalization ability of the model. DeepLesion is the largest open dataset of multi-category, lesion-level annotated clinical medical CT images published by the NIH Clinical Center so far, including 32,120 CT slices from 4,427 anonymous patients, with 1-3 lesions in each image. A total of 928,020 sheets with 32,735 lesions were included. The experimental test set selected 300 images, including 170 nodules and 130 non-nodule images. The experimental results show that the test results of the proposed method on DeepLesion are all better, the ACC, SEN and F1-score reached 92.33%, 92.68% and 0.9406, and the evaluation indicators can all reach more than 92%. To better evaluate the performance of the proposed system, the confusion matrices on the LUNA16 and DeepLesion datasets are shown in Fig. 9a, b.

By analyzing Fig. 9a, b, we can see that the proposed method is effective and can achieve about 100% correctly classified true positives and 99.3% true negative samples on LUNA16 and about 100% correct classification on DeepLesion of true positives and 85.37% of true negative samples, achieving competitive results. The above experiments verify that the proposed lung CAD system is effective for multi-center data, and the model has certain effectiveness and generalization ability.

## Discussion and conclusion

This paper proposes a lung CAD system based on the HDL model, which aims to correctly identify lung nodules and reduce the false detection and missed detection of nodules. The research mainly focuses on three aspects: feature extraction, feature fusion and recognition algorithm. First, in order to improve the feature extraction ability of the model, CBAM was embedded in VGG16 and VGG19, respectively, and AE-VGG16 and AE-VGG19 were proposed to extract features, and the more expressive nodule features were obtained. Then, through PCA dimensionality reduction and CCA fusion, fusion features with strong feature expression ability and low-dimensional characteristics are obtained. Finally, the MKL-SVM-IPSO algorithm is proposed for lung nodule identification. It uses adaptive inertia weights to speed up parameter optimization, further introduces dynamic learning factors, and adjusts the self-learning ability and collective learning ability of particles, so that the model can find the global optimum faster. optimal solution. Using the LUNA16 dataset, the ACC, SEN and F1-score reached 99.56%, 99.3% and 0.9965, respectively. The key algorithm of lung CAD system proposed in this paper has strong robustness. It can achieve good recognition accuracy and sensitivity, thus effectively avoiding false detection and missed detection of nodules.

Although the proposed lung CAD system has achieved better performance, there are still many problems to be studied. The next step will be to improve from the following three aspects:

1. The proposed lung CAD system covers feature extraction, feature dimensionality reduction, feature fusion and nodule recognition, with a wide range of steps. Model pruning techniques will be used to build lightweight networks to extract features and reduce processes. Improved feature fusion algorithm to further improve the performance of lung CAD systems.
2. Compared with natural scene images, medical images are difficult and expensive to collect, resulting in the scarcity of large-scale medical image datasets with labels. Data augmentation will be achieved through data augmentation technology to alleviate the challenge of data scarcity
3. The main limitation of deep learning lies in the dimensionality disaster and unexplainability of deep features. The above problems will be solved by fusing manual features and depth features, and designing a well-designed feature selection and feature fusion algorithm.

## Data Availability

The datasets used and analysed during the current study available from the corresponding author (X.H) on reasonable request.
